# A Cross-Sectional Study on Social Phobia Among Medical Undergraduates of Ahmedabad City

**DOI:** 10.7759/cureus.82474

**Published:** 2025-04-17

**Authors:** Sahil R Solanki, Prachi Patel, Prashant Kariya, Nupur Amin, Chintan Chaudhary, Kamleshkumar G Jain

**Affiliations:** 1 Community Medicine Department, Gujarat Cancer Society (GCS) Medical College, Hospital and Research Centre, Ahmedabad, IND; 2 Airport Health Office, Directorate General of Health Services (DGHS), Ahmedabad, IND

**Keywords:** ahmedabad city, medical students, social phobia, sports, tobacco use

## Abstract

Introduction

Medical students with social phobia are characterized by the persistent excessive fear of scrutiny, embarrassment, and humiliation in social performance; pervasive social timidity; social distress; the avoidance of some individuals; and difficulty in basic social discourses. This study aims to find the prevalence of social phobia and its association with various sociodemographic determinants among medical students in Ahmedabad city.

Methods

This cross-sectional study was conducted among Bachelor of Medicine, Bachelor of Surgery (MBBS) undergraduates from six recognized medical colleges in Ahmedabad, India. Based on a previous prevalence of social phobia (46%), the sample size was calculated as 470 using the formula n = 4pq/l² (p = 46, q = 54, and l = 4.6) and rounded to 480. From each college, 80 students (20 from each academic year) were selected using systematic random sampling.

Data were collected via personal interviews using a structured questionnaire. Part 1 captured sociodemographic details. Part 2 used the Social Phobia Inventory (SPIN) scale to categorize social phobia into five levels: none, mild, moderate, severe, and very severe.

Results

There were more women (263, 54.8%). Hostel stay (206, 42.9%) was the most common mode of accommodation, while students belonging to the upper class (369, 76.9%) were more common. Among male students, 93 (42.9%) had tobacco consumption, while among female students, 74 (28%) had tobacco consumption. A moderate grade of social phobia was most commonly seen in 136 (28.3%) medical students, followed by mild social phobia in 131 (27.3%) students. A statistically significant association was found between grades of social phobia and variables such as tobacco consumption (p ≤ 0.0001), socioeconomic class (p = 0.002), stay (p = 0.001), and involvement in sports (p ≤ 0.0001), while variables such as age, gender, and year of study did not show any statistically significant association.

Conclusion

More than half of the participants (54.8%) were female medical students. Hostel as a mode of accommodation was seen in 42.9% of the study participants, and more than three-fourths of students (76.9%) belonged to the upper class. Women had higher involvement in indoor sports as compared to men. Moderate social phobia was seen in almost one-third of the study subjects, followed by mild social phobia. Tobacco consumption, stay, socioeconomic class, and involvement in sports were significantly associated with various grades of social phobia.

## Introduction

Anxiety disorders are one of the highly prevalent common psychiatric disorders that contribute substantially to the global burden of disease and years lived with disability. Over the past few decades, it has evolved as a standalone disorder unique from the other phobias [1]. Social phobia is the second most common type of anxiety disorder and the third most common mental disorder, following depression and alcohol use disorder. Its prevalence among medical students (7%-13%) was low in Western countries compared to middle- and low-income countries (30%-90%) [2]. Diagnostic and Statistical Manual of Mental Disorders (DSM), Fifth Edition, has classified social anxiety disorder (SAD) (social phobia) under anxiety disorders, and it is defined as "a persistent fear of one or more social or performance situations in which the person is exposed to unfamiliar people or to possible scrutiny by others" [3]. Individuals experiencing social anxiety visibly struggle with social situations. They show fewer facial expressions, avert their gaze more often, and express greater difficulty initiating and maintaining conversations, compared to individuals without social anxiety [4].

Medical students with social phobia are characterized by the persistent excessive fear of scrutiny, embarrassment, and humiliation in social performance; pervasive social timidity; social distress; the avoidance of some individuals; and difficulty in basic social discourses [2]. SAD is increasingly becoming relevant in the present age of competition and pressure to perform well, especially among students [3]. Studies also indicate that younger individuals are disproportionately affected by social anxiety, with prevalence rates at around 10% by the end of adolescence, with 90% of cases occurring by age 23 [4].

The sustained social phobia without prior identification raises susceptibility to substance abuse and poor quality of life in the future. Medical students are confronted with various stressors, and the need to become accustomed suitably to achieve academic and professional success elucidates the importance of having good mental health [2]. The prevalence of social phobia among medical students was found to be 46% at Kolhapur, Maharashtra State, India [5]; 30.5% at Davangere, Karnataka State, India [6]; 41.1% at Perambalur, Tamil Nadu State, India [2]; and 11.37% at Bhavnagar, Gujarat State, India [7].

Owing to the paucity of literature regarding social phobia among medical students in Ahmedabad city, the present cross-sectional study was planned with the objectives to find the prevalence of social phobia and its association with various sociodemographic determinants among medical students in Ahmedabad city.

## Materials and methods

Study design

The present study has a cross-sectional study design.

Study setting

The research was carried out across six recognized medical colleges in Ahmedabad, Gujarat, India, encompassing students from all four academic years of the Bachelor of Medicine, Bachelor of Surgery (MBBS) program.

Study population and sampling

After taking permission from the Institutional Ethics Committee, the study was started in July 2023. The study duration was one and a half years. Based on a previous study, the prevalence of social phobia was found to be 46% [5]. Taking p as 46, q (100-p) as 54, and allowable error (l) (10% of p) as 4.6 by the formula n = 4pq/l², the sample size came to 470, which was rounded off to 480 [8].

Medical education in India has four academic years in the MBBS course. From each of the six recognized medical colleges, 80 students were selected (480/6 = 80). Further, 20 students from each academic year in each of the medical colleges were selected (80/4 = 20). These 20 students from each academic year were selected by systematic random sampling. A list of all students in each year was obtained, and every fifth student was selected until the sample size of 20 per academic year per college was obtained. For those students who refused to participate, the subsequent student was selected for the study. Those students who gave consent and were not taking any psychiatric medications were included in the study.

Data collection

Data collection was done with the selected students by personal interview. The questionnaire consisted of two parts. Part 1 included sociodemographic information about the students. The updated and modified BG Prasad classification was used to calculate socioeconomic class [9]. The second part was a scale for social phobia called Social Phobia Inventory (SPIN), which is based upon the score grading social phobia into five categories: none, mild, moderate, severe, and very severe [10].

Statistical analysis

Data entry was done in MS Excel (Microsoft Corp., Redmond, WA) and analyzed using the Statistical Package for Social Sciences (SPSS) trial version 26 (IBM Corp., Armonk, NY). Frequency, percentage, and chi-square test were used for analyzing data.

Ethical considerations

The study protocol was reviewed and approved by the Institutional Ethics Committee of Gujarat Cancer Society (GCS) Medical College, Hospital and Research Centre, Ahmedabad, India (GCSMC/EC/Project/APPROVE/2023/616). Written informed consent was obtained from all participants prior to data collection.

## Results

The sociodemographic details of the study subjects are shown in Table 1. The mean age of the students was 20.28 + 1.23 years. There were more women (263, 54.8%) as compared to men (217, 45.2%). As per the methodology, an equal number of students were selected from each academic year. Hostel stay (206, 42.9%) was the most common mode of accommodation. As per the modified BG Prasad classification, the upper class (369, 76.9%) was the most common, followed by the upper middle class (77, 16%).

**Table 1:** Sociodemographic details of the study participants (n = 480) *AICPI April 2024 = 139.4 [11] MBBS, Bachelor of Medicine, Bachelor of Surgery; AICPI, All India Consumer Price Index

Variable	Categories	Frequency	Percentage
Age (in completed years)	<20	129	26.9%
	>20	351	73.1%
Gender	Male	217	45.2%
	Female	263	54.8%
Year of study	First MBBS	120	25%
	Second MBBS	120	25%
	Third MBBS, part 1	120	25%
	Third MBBS, part 2	120	25%
Stay	Hostel	206	42.9%
	Local	201	41.9%
	Paying guest	73	15.2%
Socioeconomic class (as per the modified BG Prasad classification)^*^	Upper	369	76.9%
	Upper middle	77	16%
	Middle	13	2.7%
	Lower middle	14	2.9%
	Lower	7	1.5%

**Figure 1:**
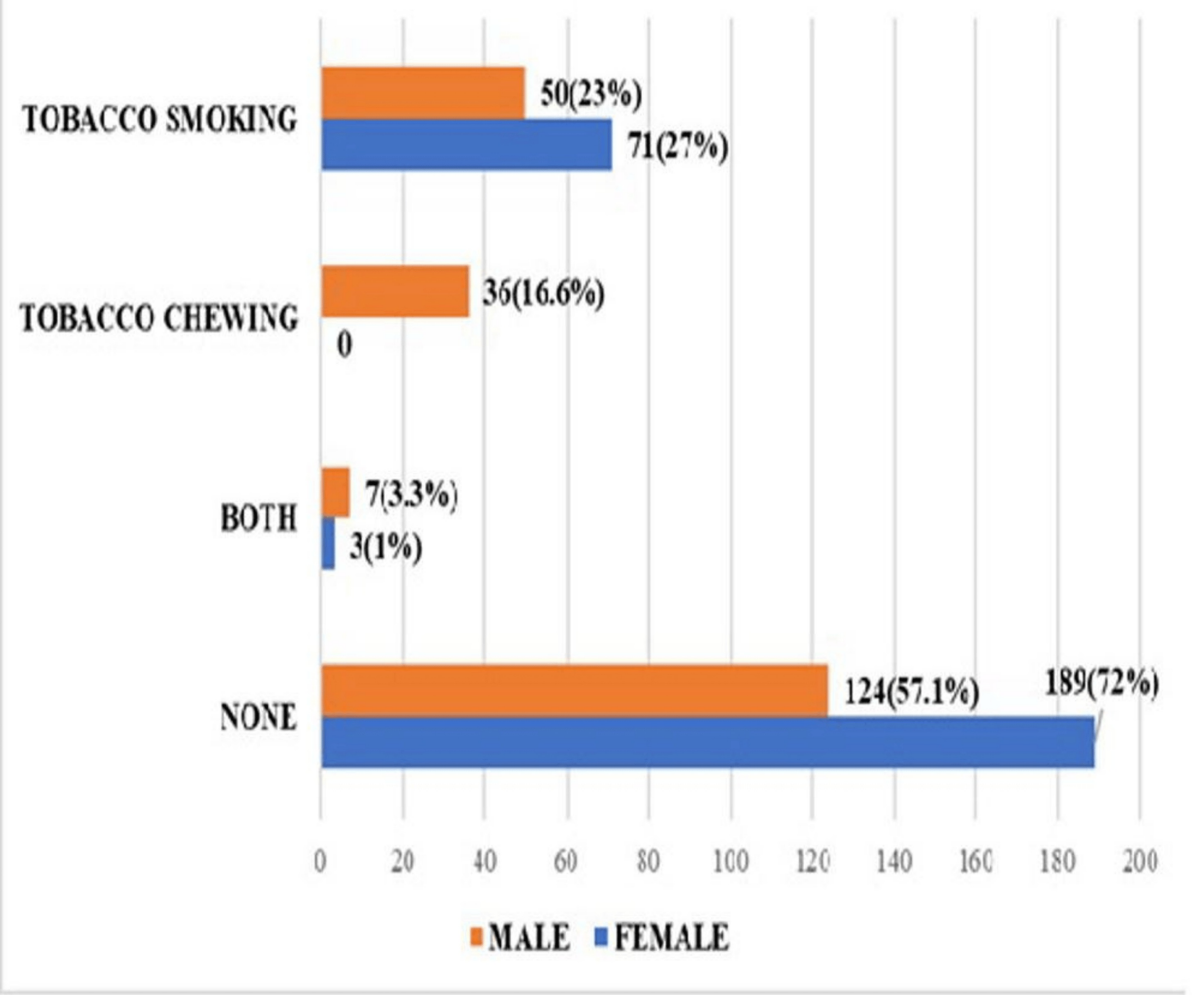
Genderwise distribution of tobacco consumption among medical students (n = 480)

Most of the study participants (313, 65%) did not have any tobacco consumption. Among male students, 93 (42.9%) were consuming tobacco, while among female students, 74 (28%) had tobacco consumption. Out of the total men (217), 50 (23%) were tobacco smokers, while 36 (16.6%) were tobacco chewers, as shown in Figure 1. Out of the total women (263), 71 (23%) were tobacco smokers, while three (1%) were both tobacco chewers and smokers.

**Figure 2:**
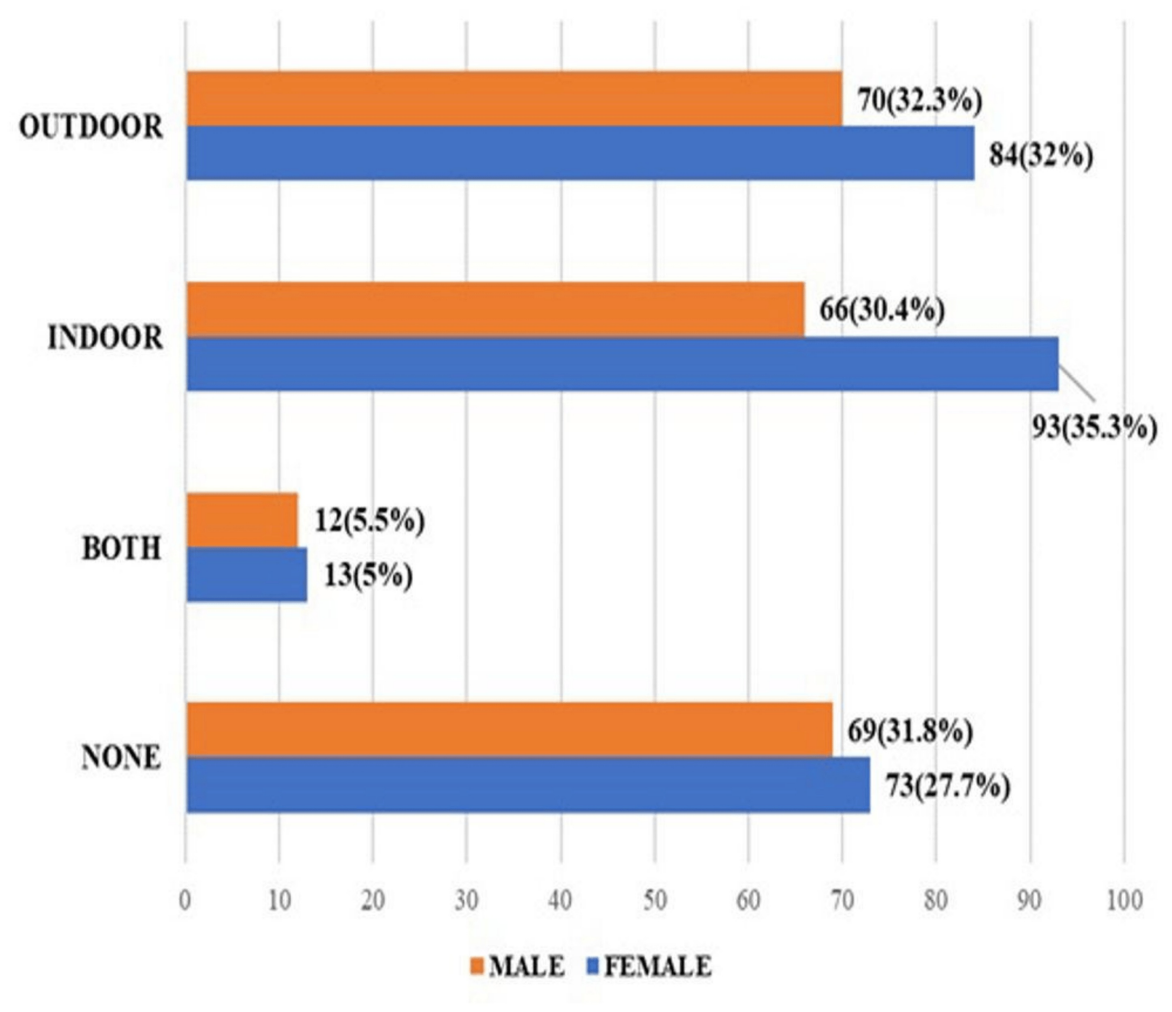
Genderwise distribution of medical students involved in sports (n = 480)

Out of the total men (217), 70 (32.3%) had outdoor sports involvement, followed by 69 (31.8%) who had no sports involvement, as shown in Figure 2. Out of the total women (263), 93 (35.3%) were into indoor sporting activity, followed by 84 (32%) who had outdoor sporting activity.

**Figure 3:**
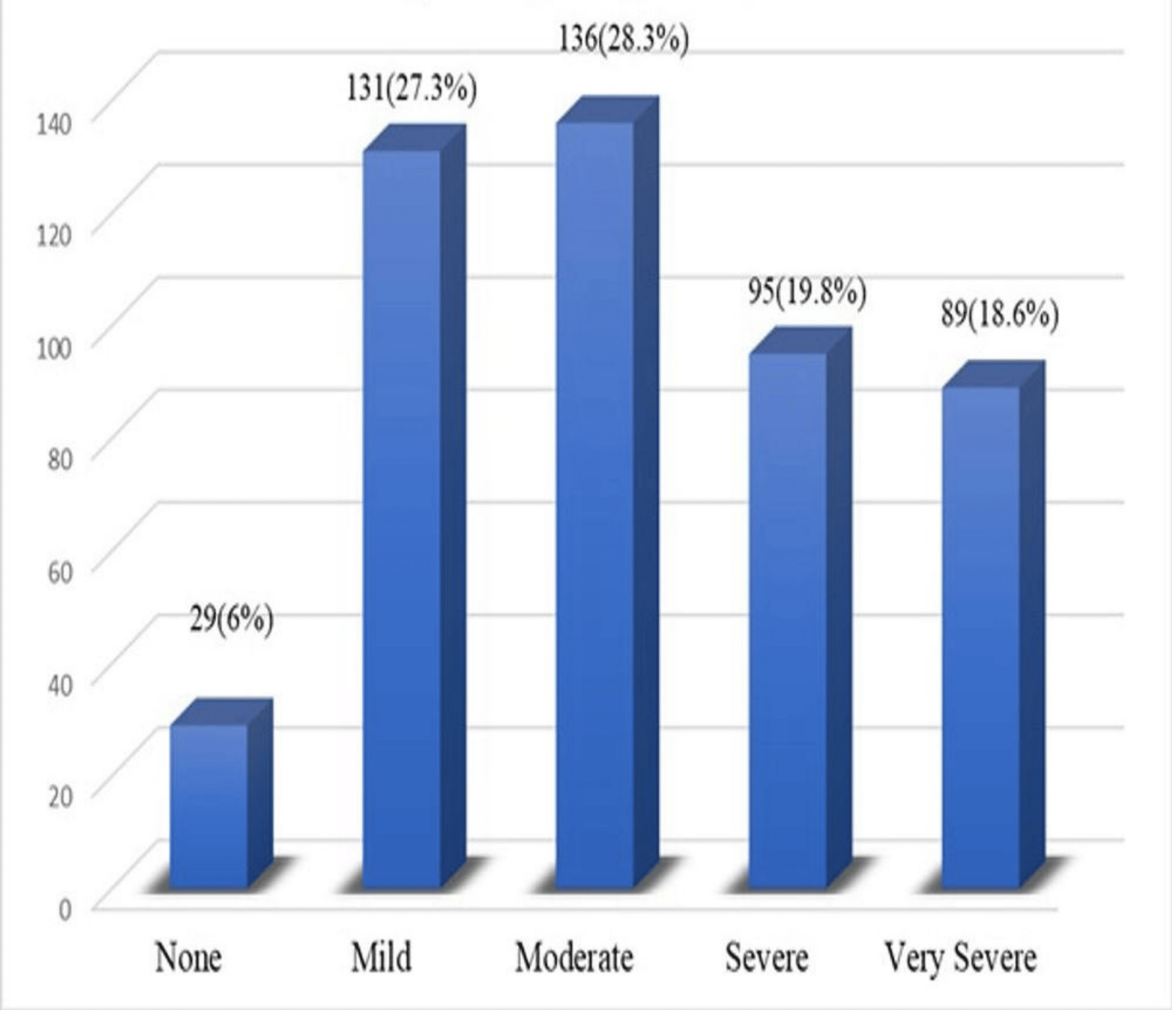
Grades of social phobia among the study participants (n = 480)

A moderate grade of social phobia was most commonly seen in 136 (28.3%) medical students, followed by mild social phobia in 131 (27.3%), as shown in Figure 3. Severe social phobia was seen in 95 (19.8%) students, while very severe social phobia was found in 89 (18.6%) medical students.

As shown in Table 2, a statistically significant association was found between grades of social phobia and variables such as tobacco consumption (p ≤ 0.0001), socioeconomic class (p = 0.002), stay (p = 0.001), and involvement in sports (p ≤ 0.0001), while variables such as age, gender, and year of study did not show any statistically significant association.

**Table 2:** Association between different variables and grades of social phobia (n = 480)

Variable	Categories	Grades of Social Phobia					Chi-Square Statistic	P-value
		None	Mild	Moderate	Severe	Very Severe		
Age	<20	6	26	41	30	26	5.915	0.206
	>20	23	105	95	65	63		
Gender	Male	17	62	62	42	34	4.153	0.386
	Female	12	69	74	53	55		
Year of study	First year	8	28	40	22	21	8.13	0.775
	Second year	4	40	28	26	21		
	Third year, part 1	9	35	33	22	24		
	Third year, part 2	8	28	35	25	23		
Tobacco consumption	None	24	106	110	60	13	168.621	<0.0001
	Tobacco chewing	2	12	10	3	9		
	Tobacco smoking	2	12	11	32	64		
	Both	1	1	5	0	3		
Socioeconomic class	Upper	23	117	96	69	64	37.467	0.002
	Upper middle	0	11	29	20	17		
	Middle	2	1	2	4	4		
	Lower middle	3	2	5	2	2		
	Lower	1	0	4	0	2		
Stay	Hostel	13	61	75	29	28	26.898	0.001
	Local	11	45	43	52	50		
	Paying guest	5	25	18	14	11		
Involvement in sports	None	0	5	16	47	74	283.327	<0.0001
	Indoor	12	32	63	39	13		
	Outdoor	17	86	47	3	1		
	Both	0	8	10	6	1		

## Discussion

The mean age of medical students was 20.28 + 1.23 years, which is closer to a study done at Bhavnagar city, India (19 ± 1.1 years) [7]. There were more female participants (54.8%) as compared to men. This finding is similar to the study carried out in Saudi Arabia, which had 56.2% female participants [12]. The present study showed hostel stay in 42.9% of students, while a study in Delhi showed 60.4% of students staying in a hostel [3]. This difference could be due to a larger number of students migrating to the national capital of India for education as compared to Ahmedabad city.

The upper socioeconomic class was most common among the study subjects (76.9%), while a study in Saudi Arabia also showed a higher number of participants (56.2%) belonging to the high-income category [12]. Tobacco consumption was found to be 42.9% among male students, which is in line with the results of the Global Adult Tobacco Survey (GATS) 2 for India [13]. For female students, tobacco consumption was found to be 28%, which is higher than the results of GATS 2 (14.2%). This difference may be due to media influence, peer pressure, and changing trends among the youth.

The present study showed almost equal participation by male and female students in indoor and outdoor gaming activities. Indoor gaming showed higher female participation (35.3%) than male participation (30.3%). This could be due to a preference for comfort, socializing opportunities, and easy accessibility.

Mild social phobia was found to be 27.3%, which is closer to the findings of the studies done at Davangere (20.6%) [6], Karnataka State, and Perambalur (21.4%) [2], Tamil Nadu State, in India. Moderate, severe, and very severe grades of social phobia were found to be 28.3%, 19.8%, and 18.6%, respectively, in the current study. These findings are higher as compared to Sudan (21.6%, 10.9%, and 9.6%) [14], Saudi Arabia (19.6%, 8.5%, and 5%) [12], and Perambalur, Tamil Nadu State, India (13.6%, 4.6%, and 1.5%) [2]. This could be due to differences in the sample size, study settings, and time frames during which the studies were conducted.

Age did not have a statistically significant association (p = 0.206) with social phobia in the present study. A similar result is also shown by the studies at Kolhapur (p = 0.384) [5], Maharashtra, and Perambalur (p = 0.597) [2], Tamil Nadu. Gender did not have a statistically significant association with social phobia (p = 0.386), which is similar to the studies at Saudi Arabia (p = 0.491) [12] and Bhavnagar city (p = 0.30) [7], India, while a study at Mangalore [15], Karnataka State, showed a significant association of gender with social phobia.

The type of accommodation or stay for medical students had a statistically significant association with social phobia (p = 0.001) in the present study, which was also shown by the study at Bhavnagar city [7], India (p = 0.01). Involvement in outdoor and indoor sports had a statistically significant association (p < 0.0001) with social phobia. This result is further supported by a recent review, which shows that sports can reduce anxiety and stress while promoting teamwork and enhancing social skills [16].

The limitations of the study include the exclusion of various sociodemographic factors such as family history of social phobia, body mass index (BMI), academic performance, internet and mobile phone usage, and family conflicts, which may influence social phobia. Additionally, the study focuses solely on the participants from Ahmedabad city. To obtain more generalized results for the state or India, a multicentric study should be conducted.

## Conclusions

More than half of the participants were female medical students. Less than half of the students were staying in a hostel, while hostel stay was a little higher than local stay. The upper socioeconomic class was seen in three-fourths of the study participants. Men had higher tobacco use than women. One-third of men and women showed participation in outdoor sports. Women had higher involvement in indoor sports as compared to men. Moderate social phobia was seen in almost one-third of the study subjects, followed by mild social phobia. Tobacco consumption, stay, socioeconomic class, and involvement in sports were significantly associated with various grades of social phobia.
